# A Broken Toothbrush in the Retropharyngeal Space in a Toddler of Sixteen Months

**DOI:** 10.1155/2015/485762

**Published:** 2015-04-28

**Authors:** Saileswar Goswami, Choitali Goswami

**Affiliations:** ^1^Department of Otolaryngology, Calcutta National Medical College, Kolkata, West Bengal 700014, India; ^2^Department of General Emergency, Medical College, Kolkata, West Bengal 700073, India

## Abstract

A toddler of sixteen months fell while brushing his teeth and his mouth hit the ground. The toothbrush broke and one-third of it including the head got impacted in his throat. The attempt of his mother to remove it with her fingers further complicated the case and the toothbrush was ultimately lodged in the retropharyngeal space at the level from C1 to C5 vertebrae. It was strongly impacted due to the presence of the bristles. The broken end of the handle was just protruding into the nasopharynx and was very difficult to locate. The first attempt of its removal was unsuccessful. The toothbrush was removed safely in the second attempt without any complication.

## 1. Introduction

We frequently get patients with foreign bodies in the aerodigestive tract but a toothbrush is a very uncommon foreign body found there. Brushing of teeth is very safe when done properly but sometimes it can lead to life-threatening complications. As toddlers have a tendency to fall, they can easily get injured while left unsupervised during toothbrushing. In the present case, a child of sixteen months was the sufferer. The head of a broken toothbrush penetrated the posterior pharyngeal wall and entered the retropharyngeal space. The reason behind reporting this case is its rarity and the difficulty it posed in its management.

## 2. Case Presentation

In a remote village, a male child of one year and four months was playfully brushing his teeth in the late morning. He fell and his mouth hit the ground. The toothbrush broke and the head along with a part of the handle remained in his throat. His mother tried to remove it with her fingers but was unsuccessful. Her attempt resulted in further injury to the throat and the child started bleeding from his mouth. He was first taken to a paediatrician who advised his parents to take him to a tertiary care hospital.

At the time of admission in the ENT department of a rural medical college, he was unable to open his mouth completely or take foods and drinks. Blood stained saliva was coming out from his mouth. His oxygen saturation was 85% and he was having mild respiratory distress. His pulse was regular and was 110/min. Respiration was also regular and was 26/min. He was conscious but very restless and was not allowing examination of his throat.

**Figure 1:**
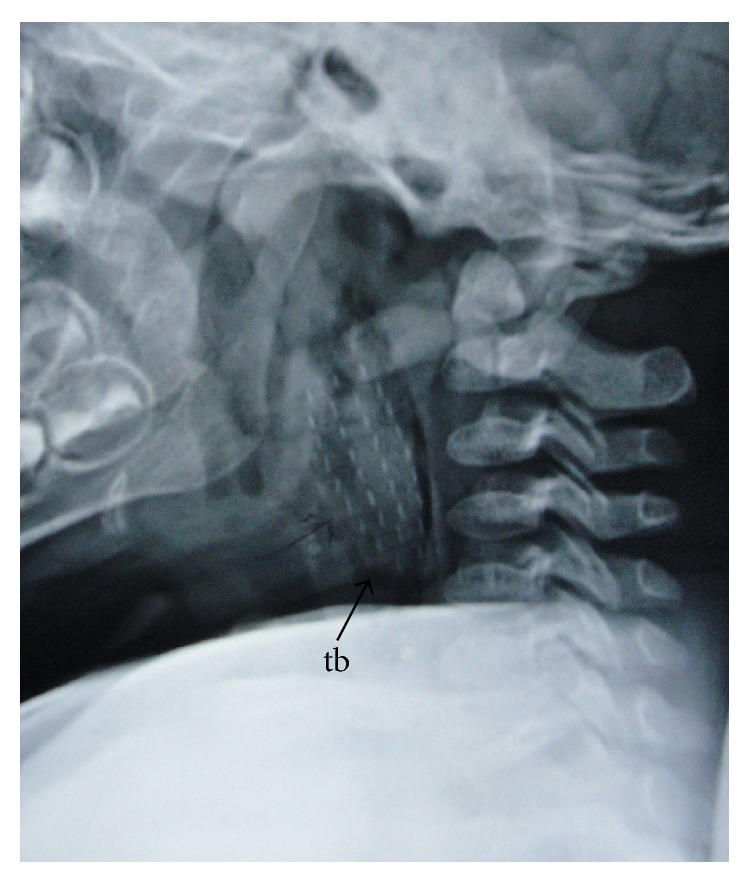
X-ray of the neck showing the head of the toothbrush (tb) with the bristles (dotted lines) in the retropharyngeal space extending from the level of C1 to C5 vertebrae.

X-ray of the neck revealed a faint radio-opaque foreign body in the prevertebral area extending from the level of C1 to C5 vertebrae (Figure 1). The airway was clearly in front of the foreign body. The shape of the foreign body and the dotted shadows in rows were in line with the head of a toothbrush with the bristles. The bristles were aligned in the coronal plane.

Treatment was started with antibiotics, analgesics, oxygen, and others. After his oxygen saturation was improved, further examination was carried out. The oral cavity was full of blood stained saliva. The mucosa of the oropharynx was edematous. There were multiple scattered superficial erosions in the mucosa of the oropharynx and the soft palate but there was no active bleeding.

The patient was taken to the operation theatre for removal of the toothbrush. He was operated under general anaesthesia with orotracheal intubation. The resident otolaryngologist, who was on duty at that time, was unable to locate the toothbrush as there was edema of the pharyngeal mucosa and no part of the toothbrush was visible on the surface. The first attempt of its removal was unsuccessful.

Conservative treatment was continued with intravenous steroids, antibiotics, and others. CT scan was advised to know the precise location of the toothbrush and its relation to the nearby critical structures. CT scan was performed after two days because of financial constraints.

**Figure 2:**
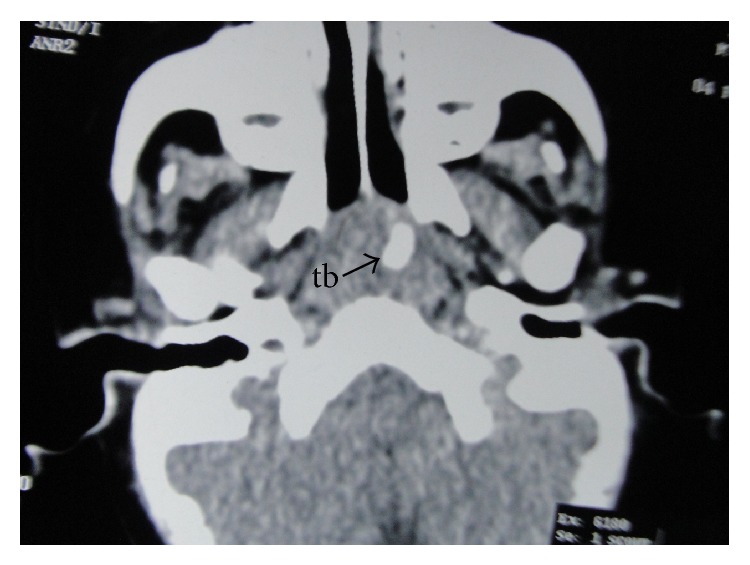
CT scan passing through the external auditory meatus, showing the handle of the toothbrush (tb) in the retropharyngeal space of the nasopharynx.

**Figure 3:**
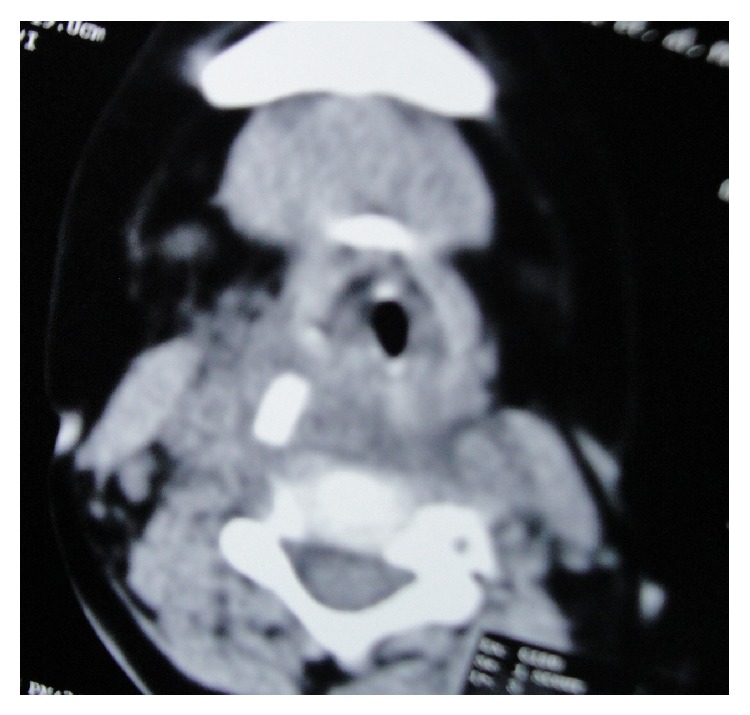
CT scan showing the lowermost part of the toothbrush in the retropharyngeal space of the hypopharynx.

**Figure 4:**
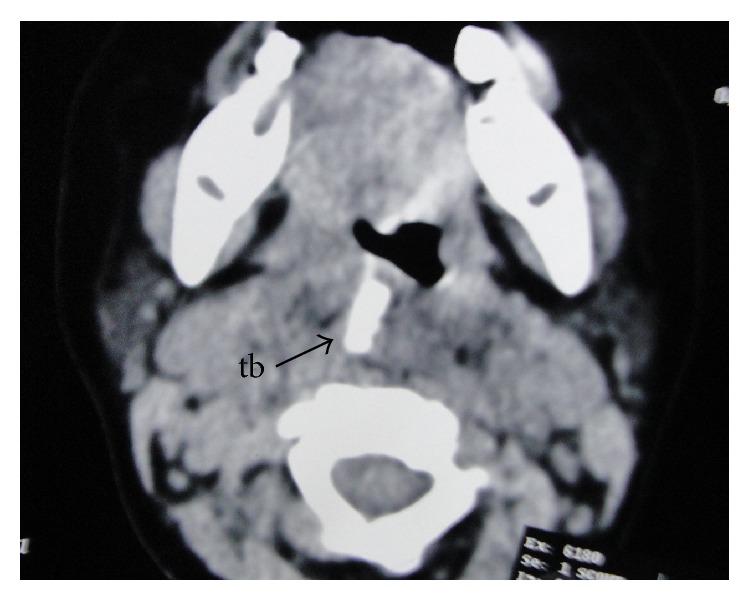
CT scan showing the toothbrush (tb) embedded in the retropharyngeal pace of the oropharynx.

**Figure 5:**
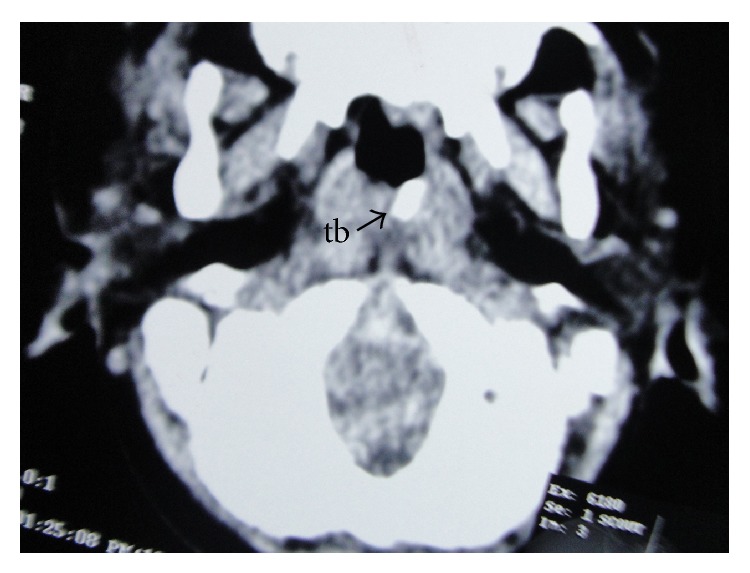
CT scan passing through the atlantooccipital joint, showing the handle of the toothbrush (tb) coming out to the surface from the retropharyngeal space of the nasopharynx.

CT scan revealed the toothbrush embedded within the prevertebral soft tissue. It was lying obliquely across the midline, extending from the roof of the nasopharynx (Figure 2) to the level of the lower border of the C5 vertebra (Figure 3). The head was directed downwards and to the right, whereas the handle was directed upwards and to the left. The head and the neck were completely embedded within the prevertebral soft tissue (Figure 4). The broken end of the handle (Figure 2) was also embedded within the prevertebral soft tissue of the nasopharynx. However, a small part of the handle below the broken end was just coming out to the surface in the nasopharynx (Figure 5). The minimum distance between the carotid artery and the toothbrush was about 1.5 cm, which was at its upper end.

It was decided to wait for two more days for the edema to subside completely. The patient was operated for the second time on the fourth day of the injury. Under general anaesthesia, a Boyle Davis mouth gag was introduced. The oral cavity and the oropharynx were cleaned by suction. The oropharynx was searched thoroughly to locate the toothbrush but it was not found. Multiple scattered superficial injuries were seen over the mucosa of the oropharynx but no entry wound was found. There was a bulging over the posterior pharyngeal wall. On palpation, the toothbrush could be felt there.

A zero degree nasal endoscope was introduced through the left nostril. After cleaning by suction, a portion of the handle was found to be protruding from the posterior wall of the nasopharynx on the left side but both ends of the toothbrush were firmly embedded within the soft tissue.

It was not possible to remove the toothbrush through the nasal route. Two rubber catheters were used for velotraction. The soft palate was retracted anteriorly to expose the nasopharynx. The portion of the handle inside the lumen of the nasopharynx was visualised directly. It was impacted within the soft tissue. The upper entry point was widened first with the help of artery forceps and the broken end of the handle was gradually disimpacted. Then the lower point was widened in the same manner. The toothbrush was then removed by gently pulling as well as slightly rotating it in either direction to free it from the surrounding soft tissue. It was done slowly under direct vision taking care to avoid injury to the surrounding structures.

**Figure 6:**
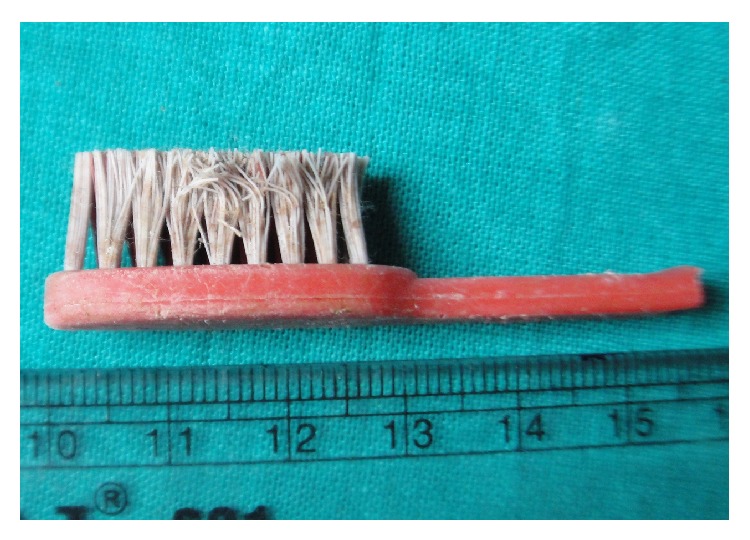
The toothbrush (5.8 cm × 1.5 cm) with sharp broken edge after removal.

After removal of the toothbrush, the wound was carefully inspected. It was 5.8 cm long and 1.5 cm wide (Figure 6). The insignificant bleeding was controlled by applying pressure. The wounds were left as they were, to be healed on their own. Ryle's tube was introduced for feeding in the postoperative period.

The patient was kept on intravenous fluid on the day of operation. Ryle's tube feeding was started from the next day. The postoperative period was uneventful and the patient recovered quickly. Ryle's tube was removed after five days and oral feeding was started. The patient was discharged seven days after the operation.

## 3. Discussion

Impalement injury and implantation of a foreign body in the oral cavity are common in young children [1] but implantation of a toothbrush in the pharynx is not common. Oza et al. [1] reported a case of implantation of a broken toothbrush medial to the ramus of the mandible in a child following an injury with a cricket ball. Moran [2] reported a case of a toothbrush embedded in the buccal soft tissues. Sagar et al. [3] reported a case of life-threatening penetrating oropharyngeal trauma in a child by a toothbrush, which had penetrated the posterolateral part of the pharyngeal wall and reached the parapharyngeal space beyond the carotid sheath and pushed it laterally at the level of the second cervical vertebra.

There are few case reports of toothbrushes in the parapharyngeal space also. Burduk [4] and Aggarwal et al. [5] also reported cases of toothbrushes in the parapharyngeal space. However, toothbrush in the retropharyngeal space is an extreme rarity. Tanaka et al. [6] reported a case of a toothbrush in the retropharyngeal space embedded beside the carotid artery, which was removed by endoscopic surgical technique. In the present case, the toothbrush was embedded obliquely across the midline, extending from the roof of the nasopharynx to the level of the lower border of the C5 vertebra.

In most of the cases, injuries caused by an unimpacted toothbrush are minor and do not require much attention. However, penetrating injuries of the oral cavity and the pharynx, with or without impaction of foreign bodies, should not be taken lightly. They can lead to massive bleeding, retropharyngeal abscess, [7] mediastinitis, [8] carotid artery thrombosis, [9] jugular vein thrombosis, and cranial nerve involvement.

Attempt to remove a penetrating pharyngeal foreign body should be done after proper investigations, which are necessary to know its precise location and relation to the nearby critical structures. CT scan should be done routinely in all cases. Angiography may be necessary to exclude major vascular injury. MRI may be necessary in selected cases. In the present case, the first attempt was unsuccessful as CT scan was unavailable due to financial constraints.

Broad spectrum antibiotics including anaerobic coverage are very important to prevent sepsis. Tetanus prophylaxis is necessary in unimmunised patients. Anti-inflammatory agents like steroids may be necessary prior to surgical intervention to reduce the edema and increase the visibility of the impacted foreign body. If the wound is old and infected, it is better to leave it open [3]. Recent, uninfected, and clean wound may be sutured. Sutures may also be necessary in case of bleeding. If the foreign body is big and close to important neurovascular structures, an external exploration [5] may be preferred. Sagar et al. [3] removed a broken toothbrush from the parapharyngeal space close to the internal carotid artery, via the oral route. In the present case, the broken toothbrush was lodged obliquely in the retropharyngeal space, extending from the nasopharynx to the C5 vertebra. Although it was very difficult to approach, the toothbrush could be removed successfully via the oral route without any complications.

## Key Messages

Brushing of teeth should be done carefully. Children should always be supervised properly by their parents to prevent accidental impaction of a toothbrush. The injury may be further aggravated by the attempt of the parents to remove it. CT scan should be done routinely in all cases of penetrating pharyngeal foreign bodies. It should be removed by experienced surgeons. Proper imaging and prompt medical care are necessary to save the patient.
